# Pharmacological inhibition of sclerostin protects bone from B‐cell acute lymphoblastic leukemia‐mediated destruction

**DOI:** 10.1002/hem3.70355

**Published:** 2026-03-31

**Authors:** Vincent Kuek, Joyce Oommen, Emanuela Ferrari, Jinbo Yuan, Aris N. Economides, Richard B. Lock, Sebastien Malinge, Rishi S. Kotecha, Laurence C. Cheung

**Affiliations:** ^1^ Leukaemia Translational Research Laboratory, WA Kids Cancer Centre, The Kids Research Institute Australia Perth Western Australia Australia; ^2^ School of Diagnostic and Therapeutic Sciences Curtin University Perth Western Australia Australia; ^3^ School of Biomedical Sciences University of Western Australia Perth Western Australia Australia; ^4^ Regeneron Pharmaceuticals Inc. Tarrytown New York United States; ^5^ Children's Cancer Institute, Lowy Cancer Research Centre, School of Clinical Medicine, UNSW Medicine & Health, UNSW Centre for Childhood Cancer Research, UNSW Sydney Sydney New South Wales Australia; ^6^ School of Medicine University of Western Australia Perth Western Australia Australia; ^7^ Department of Clinical Haematology Oncology, Blood and Marrow Transplantation, Perth Children's Hospital Perth Western Australia Australia; ^8^ Curtin Medical Research Institute Curtin University Perth Western Australia Australia

B‐cell acute lymphoblastic leukemia (B‐ALL) is the most common pediatric cancer. Current therapeutic regimens have improved 5‐year event‐free survival (EFS) rates to 90%, however clinical outcomes for high‐risk subgroups, such as BCR‐ABL1^+^ B‐ALL and relapsed ALL, remain poor.^1^ In addition, 16% of newly diagnosed children with ALL present with vertebral compression fractures.^2^ Moreover, 16% of children with ALL undergoing glucocorticoid therapy also experience a high incidence of vertebral fractures, indicating that bone health may be compromised by both leukemia progression and osteotoxicity of chemotherapy.^3^ Recently, preclinical studies have evaluated the use of osteoclast‐targeting agents for the treatment of leukemia‐induced bone loss.^4^, ^5^, ^6^ However, the use of anabolic therapies to improve bone health in children with ALL has not been investigated. Here, we evaluated the therapeutic strategy of pharmacologically restoring osteoblastic cells (OBCs) in murine B‐ALL models using a neutralizing antibody targeting sclerostin (Scl‐Ab), an osteocyte‐secreted protein which inhibits bone formation (Supplemental Methods).^7^

First, we evaluated the efficacy of Scl‐Ab treatment in the ALL‐84 patient‐derived xenograft (PDX) immunocompromised NSG mouse model, which was derived from a 14‐year‐old boy with relapsed B‐ALL.^6^ We first confirmed that ALL‐84‐bearing mice with high bone marrow (BM) disease burden (97.5 ± 0.33%) exhibited a significant reduction in the number of femoral bone‐lining cells expressing osteocalcin (OCN^+^) compared to the femurs of healthy control mice (Supporting Information S1: Figure 1A). We then treated ALL‐84‐bearing mice (BM disease burden = 11.64 ± 2.53%) with Scl‐Ab or control isotype IgG antibody (Iso‐Ab) for 2 weeks (Figure 1A). We found that Scl‐Ab significantly increased the number of femoral bone‐lining OCN^+^ cells compared to Iso‐Ab at the end of treatment (Figure 1B,C). Immunofluorescence staining identified a marginal increase in alkaline phosphatase (ALP^+^) bone cells following Scl‐Ab treatment (Supporting Information S1: Figure 2). Micro‐CT scanning revealed that Scl‐Ab treatment significantly improved both the trabecular and cortical bone parameters in ALL‐84‐bearing mice (Figure 1D,E).

**Figure 1:**
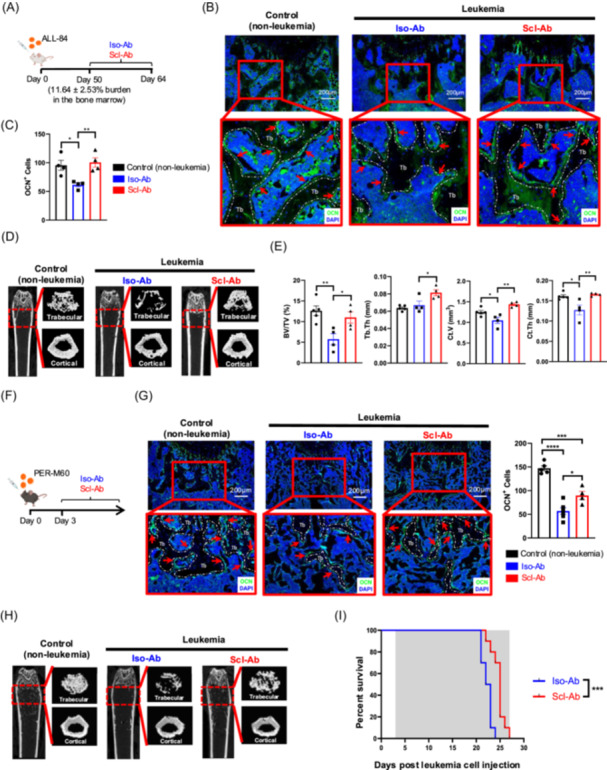
**Scl‐Ab treatment reverses B‐cell acute lymphoblastic leukemia‐induced bone loss**. **(A)** Schematic of the Scl‐Ab treatment schedule in ALL‐84 patient‐derived xenografts. Mice were treated with Scl‐Ab or Iso‐Ab for 2 weeks, starting at a bone marrow disease burden of 11.64 ± 2.53%. **(B)** Representative images followed by **(C)** quantitative analysis showing the average number of osteocalcin (OCN^+^) bone‐lining cells (per field of 0.5 mm below the growth plate) in nonleukemic control mice and ALL‐84 leukemic mice treated with Scl‐Ab or Iso‐Ab (OCN staining = green and DAPI nuclear staining = blue). Datapoints represent biological samples (*n* = 4 per group). **(D)** Representative 3‐dimensional reconstructed micro‐CT images showing the distal femur compartments of nonleukemic control mice and ALL‐84 leukemic mice treated with Scl‐Ab or Iso‐Ab. **(E)** Quantification of trabecular bone volume per tissue volume (BV/TV), trabecular thickness (Tb.Th), cortical volume (Ct.V), and cortical thickness (Ct.Th). Datapoints represent biological samples (*n* = 4–5 per group). **(F)** Schematic of the Scl‐Ab treatment schedule in PER‐M60 syngeneic mice. Mice received continuous treatment with Scl‐Ab or Iso‐Ab commencing 3 days following leukemia cell injection until they succumbed to disease. **(G)** Representative images followed by quantitative analysis showing the average number of OCN^+^ bone‐lining cells in the control (nonleukemic) and mice bearing PER‐M60 leukemia treated with Scl‐Ab or Iso‐Ab (OCN staining = green and DAPI nuclear staining = blue). Datapoints represent biological samples (*n* = 4–5 per group). **(H)** Representative 3‐dimensional reconstructed micro‐CT images showing the distal femur compartments of nonleukemic control mice and PER‐M60 leukemic mice treated with Scl‐Ab or Iso‐Ab until mice succumbed to disease. **(I)** Kaplan–Meier survival curve showing the survival of PER‐M60 leukemia‐bearing mice treated with Scl‐Ab or Iso‐Ab (*n* = 10 per group). The gray shaded areas indicate the treatment periods. Error bars represent mean ± SEM. *P < 0.05, **P < 0.01, ***P < 0.001, ****P < 0.0001.

Next, we determined whether Scl‐Ab treatment could also improve bone health during leukemia progression in a functional immune system, utilizing the PER‐M60 murine BCR‐ABL1^+^ B‐ALL syngeneic model.^8^ In this model, we again demonstrated that the femurs of leukemic mice contained lower numbers of OCN^+^ bone‐lining cells at high BM disease burden (86.8 ± 0.32%) compared to healthy control mice (Supporting Information S1: Figure 1B). We next administered Scl‐Ab or Iso‐Ab to leukemia‐bearing mice continuously, starting 3 days postleukemia transplant when the BM leukemia burden was undetectable (Figure 1F). Consistent with the ALL‐84 PDX model, Scl‐Ab treatment significantly increased the number of bone‐lining OCN^+^ cells and improved overall bone health, as shown by improved trabecular and cortical bone parameters, in BCR‐ABL1^+^ B‐ALL syngeneic mice (Figure 1G,H and Supporting Information S1: Figure 3). Immunofluorescence staining revealed a marginal increase in ALP+ bone cells following Scl‐Ab treatment (Supporting Information S1: Figure 4A–C). We have previously reported that leukemia progression also elevates the activity and number of osteoclasts.^8^ In this study, we further evaluated whether Scl‐Ab treatment affects BM osteoclasts. After 2 weeks of Scl‐Ab treatment, we found that there were no significant differences in the osteoclast parameters of leukemic mice compared to controls (Supporting Information S1: Figure 4D,F). Furthermore, we assessed whether restoring bone loss with Scl‐Ab treatment had any impact on survival of leukemia‐bearing mice. While Scl‐Ab did not directly affect the viability of leukemic cells in vitro (Supporting Information S1: Figure 4G), PER‐M60 leukemia‐bearing mice treated with Scl‐Ab demonstrated a modest but significant extension of survival compared to Iso‐Ab‐treated mice (Figure 1I).

To further confirm whether Scl‐Ab can exert beneficial effects when used in combination with conventional therapy, we treated PER‐M60‐bearing mice with Scl‐Ab or Iso‐Ab in combination with dasatinib, a tyrosine kinase inhibitor which has previously been evaluated in this leukemia model.^6^ Treatment started when BM disease burden reached 2.26 ± 0.7%, with dasatinib administered for 4 weeks and Scl‐Ab or Iso‐Ab administered during the first 2 weeks of dasatinib treatment (Supporting Information S1: Figure 5A). We found that Scl‐Ab significantly improved both the trabecular and cortical bone parameters during dasatinib treatment and that combination therapy was well tolerated with no adverse effects observed (Supporting Information S1: Figure 5B,C). In addition, we also found that Scl‐Ab treatment demonstrated a modest but significant extension of survival compared to Iso‐Ab treatment in leukemic mice undergoing dasatinib treatment (Supporting Information S1: Figure 5D). Taken together, these data support the specificity of Scl‐Ab in targeting OBCs to promote bone formation and bone health, with the additional benefit of an extension in survival either as a single agent or as combination treatment with conventional therapy.

The cellular components of the BM microenvironment are known to play a crucial role in leukemia development.^9^ We investigated the molecular changes of leukemic OBCs by performing RNA sequencing to compare the transcriptomic profiles of OBCs between healthy mice (control OBCs or C‐OBCs) and PER‐M60 BCR‐ABL1^+^ leukemia‐bearing mice (leukemia‐associated OBCs or L‐OBCs) (Supporting Information S1: Methods, Supporting Information S1: Figure 6). We identified a total of 51 differentially expressed genes (DEGs), with 19 upregulated genes and 32 downregulated genes identified in L‐OBCs compared to C‐OBCs (Figure 2A,B). Gene set enrichment analysis (GSEA) using the Kyoto Encyclopedia of Genes and Genomes (KEGG) database showed gene sets associated with necroptosis, cytokine–cytokine receptor interaction, and chemokine signaling pathway were upregulated in L‐OBCs, whilst gene sets related to osteoblast signaling pathways including AMPK, Hedgehog, Hippo, Wnt, mTOR, and TGF‐β signaling pathways were downregulated in L‐OBCs (Supporting Information S1: Figure 7A,B). Furthermore, Gene Ontology (GO) enrichment analysis of biological processes from our GSEA dataset revealed that gene sets associated with immune cell chemotaxis, chemokine‐mediated signaling pathway, and complement pathway were upregulated (Supporting Information S1: Figure 8A). Among the downregulated biological processes, gene sets associated with osteoblast activity (regulation of ossification, regulation of TGF‐β receptor signaling pathway, positive regulation of osteoblast proliferation) and osteoclast differentiation were downregulated (Supporting Information S1: Figure 8B). GO enrichment analysis of molecular function identified that gene sets associated with cytokine/chemokine activities were upregulated, and signaling pathways associated with osteoblast functions (SMAD and β‐catenin binding) were downregulated (Supporting Information S1: Figure 9A,B).

**Figure 2:**
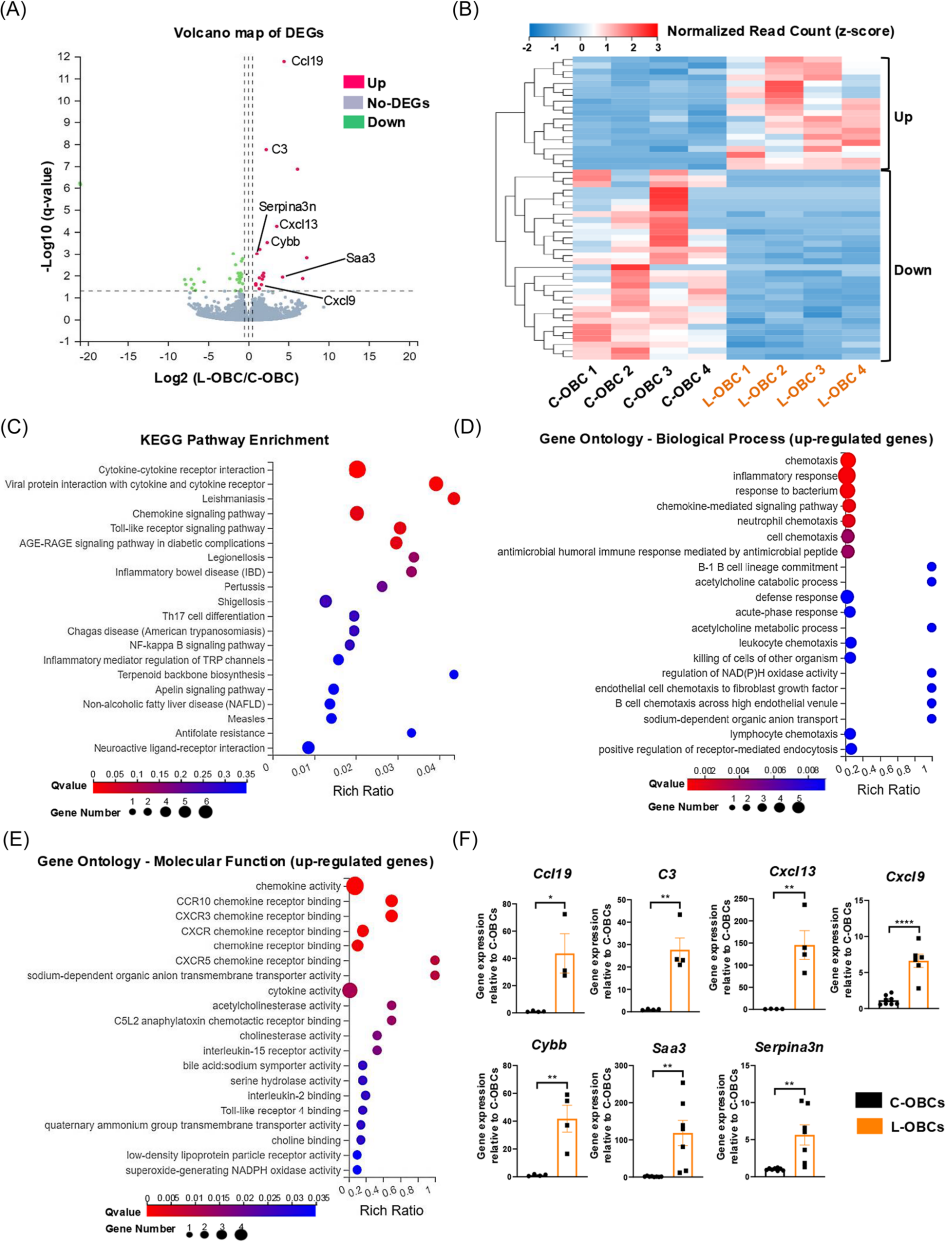
**Analysis of the osteoblastic cell (OBC) transcriptome reveals regulatory pathways associated with chemotaxis and inflammatory response are altered during the progression of BCR‐ABL1** ^**+**^ **B‐cell acute lymphoblastic leukemia**. **(A)** Volcano plot showing the upregulated and downregulated differentially expressed genes (DEGs) in leukemia‐associated OBCs (L‐OBCs) compared to control OBCs (C‐OBCs) (green = downregulated; red = upregulated, |Log2FC| ≥0.5). **(B)** Unsupervised clustering of DEGs based on normalized read counts standardized by row *z*‐score. **(C)** Kyoto Encyclopedia of Genes and Genomes (KEGG) pathway analysis of the DEGs. The top 20 most significantly enriched pathways are displayed. **(D**, **E)** Gene Ontology (GO) analysis of **(D)** biological processes and **(E)** molecular functions of the upregulated genes in L‐OBCs during leukemogenesis. The top 20 most significantly enriched pathways are displayed. All RNA sequencing data represent four biological samples per group (*n* = 4), with significance assigned by *q*‐value ≤ 0.05. **(F)** Upregulation of DEGs associated with chemotaxis and inflammatory response in L‐OBCs confirmed by real‐time quantitative polymerase chain reaction (RT‐qPCR). RT‐qPCR datapoints represent independent biological samples (*n* = 3–8 per group) to those used for RNA sequencing. Error bars represent mean ± SEM. *P < 0.05, **P < 0.01, ****P < 0.0001.

Consistent with our GSEA data, annotation of genes using the KEGG pathway database revealed significantly enriched transcriptional signatures related to cytokine‐cytokine receptor interaction (*q*‐value = 0.0069) and to the chemokine signaling pathway (*q*‐value = 0.038) (Figure 2C). Amongst upregulated DEGs, GO enrichment analysis demonstrated biological processes including chemotaxis (*q*‐value = 0.0016), inflammatory response (*q*‐value = 0.0018), chemokine‐mediated signaling pathway (*q*‐value = 0.002), neutrophil chemotaxis (*q*‐value = 0.002), cell chemotaxis (*q*‐value = 0.004), and acute‐phase response (*q*‐value = 0.009) as being the most significantly enriched signatures (Figure 2D). Furthermore, GO enrichment analyses of upregulated DEGs revealed “chemokine activity” as being the most significantly enriched molecular function (*q*‐value = 9.3 × 10^−6^), thus validating our GSEA findings (Figure 2E). We identified *Ccl19*, *C3*, *Cxcl13*, *Cxcl9*, *Cybb, Saa3*, and *Serpina3n* as upregulated DEGs responsible for the inflammatory response (|Log2FC| ≥1, *q*‐value ≤ 0.05), further validated by real‐time quantitative polymerase chain reaction (Figure 2A,F). Finally, while in vitro exposure to murine CCL19 and SAA3 recombinant proteins did not directly alter the number of viable PER‐M60 leukemic cells (data not shown), murine CXCL9 and CXCL13 recombinant proteins promoted an increase in the number of viable PER‐M60 leukemia cells (Supporting Information S1: Figure 10A,B). These results indicate that B‐ALL may impair the proliferation/function of OBCs and shift their molecular signature to a pro‐inflammatory state within the BM microenvironment, favoring disease progression.

Skeletal abnormalities and increased fracture risk are known to be significant comorbidities which can negatively affect the immediate and long‐term development of children with ALL.^3^ Recent research suggests that targeting osteoclasts not only promotes bone health but also improves survival in B‐ALL mouse models.^6^ However, the effectiveness of therapeutic agents targeting OBCs in B‐ALL remains elusive. Here, we showed that Scl‐Ab treatment could reverse bone loss in two high‐risk B‐ALL mouse models. While the Scl‐Ab did not have a direct effect on the viability of leukemic cells, we observed a survival advantage when leukemia‐bearing mice were treated with Scl‐Ab. A similar observation was reported in acute myeloid leukemia, where increased OBC numbers were associated with suppressed leukemia progression.^10^ Our data also showed that combining Scl‐Ab therapy with dasatinib extended survival while concurrently improving bone health. Others have also shown that Scl‐Ab can exert additive antitumor effects when used in combination with carfilzomib to treat mice bearing multiple myeloma, and that Scl‐Ab does not negatively impact the efficacy of conventional therapeutics in vitro.^11^, ^12^ Future studies should evaluate whether combining Scl‐Ab with other conventional chemotherapeutic agents, such as vincristine, dexamethasone, and l‐asparaginase, can prevent bone loss in different ALL subtypes. The mechanistic insight of survival extension by Scl‐Ab requires further exploration and future studies to explore this therapeutic strategy for other high‐risk subtypes of ALL are also warranted. Notably, favorable results from phase III clinical trials have led to FDA approval of humanized Scl‐Ab (romosozumab) for use in osteoporotic post‐menopausal women associated with high fracture risk.^13^ Promisingly, a preclinical study has found that Scl‐Ab treatment could confer bone health benefits without affecting bone quality in 4‐week old mice with osteogenesis imperfecta, suggesting that promoting osteoblast function via Scl‐Ab treatment does not adversely impact bone growth.^14^ Several clinical studies are also currently underway to evaluate Scl‐Ab in children and adolescents with osteogenesis imperfecta (NCT04545554, NCT05972551). Outcomes from these studies could provide further rationale for investigating potential of Scl‐Ab to reduce fracture risks, decrease bone morbidity, and enhance quality of life in pediatric patients with hematological malignancies.

The ability of malignant hematopoietic cells to remodel the healthy BM microenvironment into a “safe sanctuary” that contributes to disease development and dysfunctional hematopoiesis is well documented in myeloid malignancies.^15^ For lymphoid malignancies, we have reported that the BM microenvironment is extensively remodeled in B‐ALL, with increased osteoclast bone resorption and decreased osteoblast numbers contributing to pathological bone loss.^8^ Despite this, the mechanism which mediates the crosstalk between leukemic cells and OBCs remain unclear. Our transcriptomic data revealed that L‐OBCs upregulated genes encoding proteins associated with chemotaxis and inflammatory response. In particular, expression of *cxcl13* and *cxcl9* were upregulated by L‐OBCs. We further confirmed that CXCL13 and CXCL9 recombinant proteins promoted an increase in the number of PER‐M60 B‐ALL cells in vitro. Indeed, CXCL13 has been reported as a critical factor in promoting proliferation and migration of *BCR‐ABL1* ^*+*^ B‐ALL cells.^16^ On the other hand, studies have shown that CXCL9 is elevated in the bone marrow of patients with B‐ALL, and that it plays a critical role in the inhibition of angiogenesis‐osteogenesis coupling, likely contributing to leukemia cell survival and bone loss.^17^, ^18^ Our transcriptomic data also revealed molecular signatures associated with impaired osteoblast function and apoptosis in L‐OBCs. For instance, *Serpina3n* is a compelling suppressor of osteoblast differentiation and thus may contribute to the bone loss phenotype in B‐ALL.^19^ Moreover, *CYBB* encodes NADPH oxidase 2 (*NOX2*), which is involved in the synthesis of reactive oxygen species, a critical catalyst of oxidative stress that can promote osteoblast apoptosis.^20^ Taken together, this transcriptomic data supports the hypothesis that B‐ALL remodels the BM niche into a pro‐leukemia BM microenvironment which could impair the function of OBCs. Future studies should validate the role of these proteins in leukemia function and further evaluate their potential as therapeutic targets in B‐ALL.

In conclusion, our preclinical study has shown that targeting OBCs is a promising therapeutic strategy to restore ALL‐induced bone loss. Future clinical trials to investigate the combination of conventional chemotherapy with bone anabolic therapies such as Scl‐Ab are warranted in children with high‐risk B‐ALL who are at increased risk of developing incident fractures.

## AUTHOR CONTRIBUTIONS

**Vincent Kuek**: Conceptualization; methodology; formal analysis; visualization; writing—original draft; writing—review and editing; investigation. **Joyce Oommen**: Methodology; writing—review and editing. **Emanuela Ferrari**: Methodology; writing—review and editing. **Jinbo Yuan**: Methodology; writing—review and editing. **Aris N. Economides**: Writing—review and editing; resources. **Sebastien Malinge**: Methodology; writing—review and editing; formal analysis. **Rishi S. Kotecha**: Conceptualization; investigation; writing—review and editing; funding acquisition; supervision; formal analysis; resources; project administration. **Laurence C. Cheung**: Conceptualization; investigation; writing—original draft; writing—review and editing; supervision; formal analysis; project administration; resources; funding acquisition.

## CONFLICT OF INTEREST STATEMENT

ANE is an employee of Regeneron Pharmaceuticals Inc. All other authors declare that they have no conflicts of interest.

## ETHICS STATEMENT

All experimental studies were approved by the Animal Ethics Committee, The Kids Research Institute Australia (AEC #330 and #P2176).

## FUNDING

This work was supported by grant 1184963 awarded through the 2019 Priority‐driven Collaborative Cancer Research Scheme, which was co‐funded by Cancer Australia, Cure Cancer, and the Leukaemia Foundation of Australia. RSK is supported by a Fellowship from the National Health and Medical Research Council of Australia (NHMRC APP2033152) and by the Western Australia Future Health Research and Innovation (FHRI) Fund. RBL was supported by a Fellowship from the NHMRC (APP1157871). Open access publishing facilitated by Curtin University, as part of the Wiley ‐ Curtin University agreement via the Council of Australasian University Librarians.

## Supporting information

Hemasphere Letter to the Editor Supplemental Methods.

Hemasphere Supplemental Figures and Legends.

## Data Availability

The data that support the findings of this study are openly available in Gene Expression Omnibus at https://www.ncbi.nlm.nih.gov/geo/, reference number GSE298870. Raw sequencing data is available via the Gene Expression Omnibus (GEO) database under the accession number GSE298870.
