# Supplementary material for: Pharmacological inhibition of sclerostin protects bone from B‐cell acute lymphoblastic leukemia‐mediated destruction
Source: Hemasphere. 2026 Mar 31;10(4):e70355. doi: 10.1002/hem3.70355 (PMC13103727; doi:10.1002/hem3.70355)
Supplement: Supplementary file 1 — Hemasphere Letter to the Editor Supplemental Methods. [file HEM3-10-e70355-s001.docx]

**Supplemental Methods**

Pharmacological inhibition of sclerostin protects bone from B-cell acute lymphoblastic leukemia-mediated destruction

**Culture of PER-M60 leukemic cells**

Media and reagents for cell culture were purchased from Thermo Fisher Scientific (USA). Fetal calf serum was purchased from CellSera (Australia). Briefly, a mouse B-cell acute lymphoblastic leukemia (B-ALL) cell line (PER-M60) carrying murine stem cell virus (MSCV) vector co-expressing human BCR-ABL1 (p185) and mCherry (MSCV-BCR-ABL1-IRES-mCherry) was cultured and maintained in complete RPMI media (i.e., RPMI supplemented with 10% fetal calf serum (FCS), 1x penicillin/streptomycin and 2mM L-glutamine) in accordance with our previously published protocol.(1) PER-M60 cells were grown and maintained at 37°C and 5% CO_2_. For recombinant protein experiments, PER-M60 cells were seeded in 24 well plates at a density of 1 x 10^4^ cells per well, in complete RPMI medium. Cells were treated with mouse recombinant CXCL13 or CXCL9 at 200ng/mL for 9 days, with the recombinant proteins replenished on day 3 and 6. Untreated cells were used as controls. Cells were stained by ViaStain™ AOPI staining solution, loaded onto Cellometer™ SD100 slides and counted using the Cellometer K2 Fluorescent Cell Counter (Revvity Health Sciences Inc, USA).

**Generation and *in vivo* treatment of B-ALL mouse models**

Seven to 10-week old C57BL/6J and NOD-scid IL2Rgammanull (NSG) mice were purchased from the Animal Research Centre, Perth. All animals were housed under pathogen-free conditions at the Bioresources facilities of The Kids Research Institute Australia. For the patient-derived xenograft mouse model, non-irradiated NSG mice were intravenously injected with relapsed B-ALL (ALL-84) cells derived from a 14-year-old boy, as described previously.(2) ALL-84-bearing mice were treated with either 25mg/kg of a neutralizing antibody targeting sclerostin (Scl-Ab) or control isotype IgG antibody (Iso-Ab) twice weekly via subcutaneous injection, starting at a disease burden of 11.64±2.53% in the bone marrow (BM) for a duration of 2 weeks in accordance with published protocols.(3, 4). Age-matched, non-leukemic control mice did not receive any treatments. The syngeneic BCR-ABL1^+^ B-ALL mouse model was generated by transplanting PER-M60 leukemic cells into non-irradiated C57BL/6J mice as described previously.(1, 2) PER-M60 mice were treated with either 25mg/kg of Scl-Ab or Iso-Ab twice weekly via subcutaneous injection, starting 3 days post-leukemia cell injection. Treatment was administered continuously until the designated timepoints or when mice succumbed to disease. For combination therapy, PER-M60 mice were treated with dasatinib (10mg/kg twice daily via oral gavage for 28 days) and Iso-Ab or Scl-Ab (25mg/kg twice weekly for 2 weeks) when the disease burden in the BM was 2.26 ± 0.7%. Age-matched, non-leukemic control mice did not receive any treatments. Bones, spleen and blood were collected for downstream experiments.

All antibodies used for treatment were provided by Regeneron (USA). All experimental studies were approved by the Animal Ethics Committee, The Kids Research Institute Australia (AEC #330 and #P2176).

**Micro-computed tomography (micro-CT) analysis**

Femurs of mice were fixed in 4% paraformaldehyde (PFA) in phosphate buffered saline (PBS) for 2 days. Following fixation, bone samples were immobilized in 2ml tubes wrapped in PBS-moist tissue and loaded into the bed of a Skyscan 1176 micro-CT scanner (Bruker, Belgium). Distal femurs were scanned using the following parameters: 50kV, 500μA, 1000ms, 0.5mm Al filter, 9-μm pixel resolution, rotation step of 0.4°, frame averaging of 2.(1, 5) Reconstruction of scans (from midshaft to distal articular surface) was performed using NRecon software (Bruker, Belgium) at a constant threshold value. Orientations were corrected using DataViewer software (Bruker, Belgium). Both trabecular and cortical bone parameters were analyzed at the metaphyseal region beginning 0.5mm below the bottom of the growth plate and extending 1 mm proximally using CTAn software (Bruker, Belgium). Three-dimensional trabecular and cortical bone images were generated using CTVol software (Bruker, Belgium). All micro-CT procedures and data analyses were performed at the Centre for Microscopy, Characterisation and Analysis (CMCA), University of Western Australia.

**Tartrate-resistant acid phosphatase (TRAP) histomorphometry**

To prepare paraffin-embedded sections, bones were fixed in 4% PFA in PBS and incubated at 4°C for 48 hours, followed by 8 days of decalcification in 10% EDTA with gentle agitation at 4°C.(1, 2) Bones were then dehydrated via ethanol, immersed in xylene and embedded with paraffin. Sections of 5μm thickness were prepared, followed by dewaxing and staining with TRAP, as previously described.(5) Images were captured using a Nikon Eclipse Ci Microscope fitted with a DS-L3 microscope control unit (Nikon, Japan). Osteoclast surface per bone surface (OC.S/B.S) and number of osteoclasts per bone surface (OC.N/B.S) were quantified using Quantitative Pathology & Bioimage Analysis (QuPath) software.(5)

**Immunofluorescence staining**

To prepare bone slides for immunofluorescence staining, PFA-fixed bones were fixed, decalcified, dehydrated and paraffin-embedded as previously described.(1, 2) On the day of staining, sections of 5μm thickness were de-paraffinized and rehydrated, followed by antigen retrieval in 80°C sodium citrate buffer for 20 minutes. Sections were then washed in 1X Tris-buffered saline (TBS) for 10 minutes, blocked in 3% H_2_O_2_ in TBS for 10 minutes, and rinsed twice in 1X TBS/0.01% Tween-20 (1X TBST) for 5 minutes. Sections were then blocked in 10% bovine serum albumin or donkey serum (Sigma Aldrich, Australia) for 2 hours, followed by staining with primary antibody at 4°C in a humidified chamber overnight. The next day, sections were washed and stained with secondary antibody for 1 hour. After secondary antibody staining, sections were washed in 1X TBST and mounted with Fluoromount-G™ Mounting Medium, with DAPI (Thermo Fisher Scientific, USA). Fluorescence was visualized using a Nikon Ts2R Fluorescent Inverted Microscope fitted with a DS-Qi2 microscope control unit (Nikon, Japan). The number of fluorescent cells within a defined region of interest (0.5mm below the growth plate) was quantified using QuPath software.(5) The following antibodies and dilutions were used: primary rabbit anti-mouse osteocalcin IgG antibody (1:500, ab93876), primary goat anti-mouse alkaline phosphatase/ALPL IgG antibody (1:100, AF2910), secondary donkey anti-rabbit IgG antibody conjugated with Alexa Fluor® 488 (1:200, ab150073), secondary donkey anti-goat IgG antibody conjugated with Cy3® (1:200, ab6949). All antibodies used for immunofluorescence staining were purchased from R&D Systems or Abcam, USA.

**Isolation and staining of primary osteoblastic cells (OBCs) from murine long bones**

PER-M60 cells were resuspended in PBS and intravenously injected into non-irradiated C57BL/6J mice aged between 7 to 10-weeks old as previously described.(1, 2) Age-matched, non-leukemic mice were injected with PBS as control. Following disease development, mice were euthanized when disease burden in the BM reached 30-80% leukemic blasts (defined as cells expressing mCherry marker), typically around 2-3 weeks after leukemia transplantation. Following euthanasia, primary OBCs were isolated from murine long bones in accordance with previously published protocols with modifications.(6) Briefly, tibias and femurs were excised from leukemic mice and control mice following euthanasia. To isolate primary OBCs, BM was flushed out of the medullary cavity with 5% FCS/PBS. Long bones were cut into small fragments and washed thrice with PBS to remove BM remnants. Cleared bone fragments were then incubated at 37°C for 60 minutes under agitation in the presence of bone digestion media, which consists of 1.5mg/ml Collagenase Type 4 (Worthington Biochemical Corp, USA), 0.1mg/ml DNase I (Sigma Aldrich, Australia) and 10% FCS in PBS.(7) Post-digest supernatants were filtered through sterile 100μm cell strainers and collected. The remaining bone fragments were crushed gently with a mortar and pestle and flushed into the supernatants 6 times to increase the yield of OBCs. Combined BM stromal cell suspensions were treated with Red Blood Cell Lysis Buffer (BD Biosciences, USA) for 5 minutes at room temperature, and then resuspended in 5% FCS/PBS. All cells were stained with Fixable Viability Stain 700 (BD Biosciences, USA) for 15 minutes at room temperature to exclude non-viable cells prior to further antibody staining.

To stain for OBCs, stromal-derived cells were first stained with CD45-PerCP-Cy5.5 and Ter119-PerCP-Cy5.5 to exclude hematopoietic cells. Leukemia cells were also excluded by the mCherry^+^ marker. Subtypes of BM stromal cells were then stained with CD31-FITC to exclude endothelial cells, followed by Sca-1-BV510 and CD51-BV421 staining to detect OBCs (identified as CD45^-^Ter119^-^CD31^-^Sca-1^-^CD51^+^).(6) OBCs derived from the long bones of non-leukemic control mice were denoted control OBCs (C-OBCs), and OBCs derived from the long bones of leukemic mice were denoted leukemia-associated OBCs (L-OBCs). Flow cytometry was performed using a BD Fortessa and fluorescence-activated cell sorting (FACS) was performed using a BD FACSAria. Flow cytometry data was analyzed using FlowJo V10.5.3 software (BD Biosciences, USA). All antibodies used for flow cytometry analyses were purchased from BD Biosciences, USA.

**RNA sequencing of OBCs**

C-OBCs and L-OBCs were isolated, stained and sorted into a purified population via FACS using a protocol that we have described previously.(7, 8) Due to low cell yields, long bones from 4 mice (8 femurs and 8 tibias) were pooled and processed, yielding an average of ~38000 cells per sample following sorting. RNA was extracted from the sorted OBCs using the RNeasy Micro Kit (Qiagen, Germany). Samples were sent to BGI, Hong Kong for further sequencing. The total mass of RNA ranged from 6.6μg to 25.3μg, with a RNA integrity number ranging from 7.5 to 9.1 as assessed by an Agilent 2100 BioAnalyzer (Agilent, USA). Amplification of total RNA and cDNA synthesis were performed using the SMART-Seq v4 Ultra Low Input RNA Kit (Takara Bio, Japan). DNA circularization was performed to generate DNA nanoballs prior to 100bp paired-end RNA sequencing using the BGISEQ-500 platform (BGI, Hong Kong).

Data processing and analysis was performed by BGI, Hong Kong, using a similar approach to our previously published studies.(7, 8) Quality control and read filtering was performed to remove low quality reads, reads with adaptor sequences and reads with high levels of N base (>5%) using SOAPnuke software v1.5.2 (BGI, Hong Kong). Filtered clean reads ranged between 66-69 million per sample. Bowtie2 software (v2.2.5) was used to map clean reads to the reference genome of *Mus musculus* ([GCF_000001635.26_GRCm38.p6](https://www.ncbi.nlm.nih.gov/assembly/GCF_000001635.26)). RSEM software (v1.2.8) was used to calculate the gene expression level of each sample. Differentially expressed genes between C-OBCs and L-OBCs were detected by the DESeq2 method (|Log2FC|≥0.5, q-value≤0.05). For Gene Set Enrichment Analysis, a |normalized enrichment score (NES) |≥ 1 or ≤ -1, nominal p-value ≤ 0.05 and false discovery rate q-value ≤ 0.25 were used as threshold values. Kyoto Encyclopedia of Genes and Genomes pathway and Gene Ontology enrichment analyses were performed by Dr Tom software, as previously described. (7, 8) Four pseoduogenes (LOC100043921, Gm2457, Gm2564, Gm12407) withdrawn from the National Centre for Biotechnology Information or Mouse Genome Informatics databases were excluded from RNA sequencing analyses. Raw sequencing data are available via the Gene Expression Omnibus database under the accession number GSE298870.

**TaqMan real-time quantitative polymerase chain reaction (RT-qPCR)**

RNA from primary OBCs were extracted using the RNeasy Plus Mini Kit (Qiagen, Germany) according to the manufacturer’s instructions. Total RNA was quantified using the Qubit™ RNA BR Assay Kit on a Qubit 4 fluorometer (Thermo Fisher Scientific, USA). cDNA was synthesized from total RNA using SuperScript™ IV VILO™ Master Mix (Thermo Fisher Scientific, USA) according to the manufacturer’s instructions. To perform RT-qPCR, a mastermix containing cDNA, 1X TaqMan Fast Advanced Master Mix (Thermo Fisher Scientific, USA) and 1X TaqMan assay (Thermo Fisher Scientific, USA) in a final volume of 10μl was prepared for each sample. RT-qPCR was performed at 50°C for 2 minutes and 95°C for 2 minutes, followed by 40 cycles of 95°C for 1 second and 60°C for 20 seconds in a QuantStudio™ 7 Flex System (Thermo Fisher Scientific, USA). The following murine TaqMan probes were used: *Ccl19* (Mm00839967_g1), *Cxcl13* (Mm04214185_s1), *Cybb* (Mm01287743_m1), *Serpina3n* (Mm00776439_m1), *Saa3* (Mm00441203_m1), *C3* (Mm01232779_m1), *Cxcl9* (Mm00434946_m1) and *Hprt* (Mm03024075_m1). Relative gene expression quantification was performed using the 2^-ΔΔ^*^CT^* method.(9)

### **Statistical analysis**

Statistical analyses were carried out using Prism software, version 8.4.0 (GraphPad, USA), with software-recommended tests used for statistical assessment. A two-tailed unpaired Student’s t-test was used to compare the mean between two groups. One-way analysis of variance (ANOVA) with Tukey/Sidak’s correction was used to evaluate comparisons among multiple groups. For survival studies, Kaplan-Meier curves were generated to illustrate time to humane endpoint, stratified by group. The log-rank test was used to assess the median time to endpoint between different groups. All values are represented as mean ± standard error of the mean. A p value <0.05 was considered statistically significant.

### **References**

1. Cheung LC, Tickner J, Hughes AM, Skut P, Howlett M, Foley B, Oommen J, Wells JE, He B, Singh S, Chua GA, Ford J, Mullighan CG, Kotecha RS, Kees UR. New therapeutic opportunities from dissecting the pre-B leukemia bone marrow microenvironment. Leukemia. 2018 Nov;32(11):2326-2338. Epub 2018/05/10. doi:10.1038/s41375-018-0144-7. Cited in: Pubmed; PMID 29740160.

2. Kotecha RS, Trinder SM, Hughes AM, Mullin BH, Rashid S, Yuan J, Xu J, Duncan O, Skut P, Chua GA, Singh S, Oommen J, Lock RB, Kees UR, Malinge S, Kuek V, Cheung LC. Targeting osteoclasts for treatment of high-risk B-cell acute lymphoblastic leukemia. Blood Cancer J. 2025 Feb 27;15(1):25. Epub 2025/02/28. doi:10.1038/s41408-025-01239-3. Cited in: Pubmed; PMID 40016188.

3. Yee CS, Xie L, Hatsell S, Hum N, Murugesh D, Economides AN, Loots GG, Collette NM. Sclerostin antibody treatment improves fracture outcomes in a Type I diabetic mouse model. Bone. 2016 Jan;82:122-34. Epub 2015/05/09. doi:10.1016/j.bone.2015.04.048. Cited in: Pubmed; PMID 25952969.

4. Donham C, Chicana B, Robling AG, Mohamed A, Elizaldi S, Chi M, Freeman B, Millan A, Murugesh DK, Hum NR, Sebastian A, Loots GG, Manilay JO. Sclerostin Depletion Induces Inflammation in the Bone Marrow of Mice. Int J Mol Sci. 2021 Aug 24;22(17). Epub 2021/09/11. doi:10.3390/ijms22179111. Cited in: Pubmed; PMID 34502021.

5. Chen K, Liao S, Li Y, Jiang H, Liu Y, Wang C, Kuek V, Kenny J, Li B, Huang Q, Hong J, Huang Y, Chim SM, Tickner J, Pavlos NJ, Zhao J, Liu Q, Qin A, Xu J. Osteoblast-derived EGFL6 couples angiogenesis to osteogenesis during bone repair. Theranostics. 2021;11(20):9738-9751. Epub 2021/11/25. doi:10.7150/thno.60902. Cited in: Pubmed; PMID 34815781.

6. Schepers K, Pietras EM, Reynaud D, Flach J, Binnewies M, Garg T, Wagers AJ, Hsiao EC, Passegue E. Myeloproliferative neoplasia remodels the endosteal bone marrow niche into a self-reinforcing leukemic niche. Cell Stem Cell. 2013 Sep 5;13(3):285-99. Epub 2013/07/16. doi:10.1016/j.stem.2013.06.009. Cited in: Pubmed; PMID 23850243.

7. Hughes AM, Kuek V, Oommen J, Chua GA, van Loenhout M, Malinge S, Kotecha RS, Cheung LC. Characterization of mesenchymal stem cells in pre-B acute lymphoblastic leukemia. Front Cell Dev Biol. 2023;11:1005494. Epub 2023/02/07. doi:10.3389/fcell.2023.1005494. Cited in: Pubmed; PMID 36743421.

8. Hughes AM, Kuek V, Oommen J, Kotecha RS, Cheung LC. Murine bone-derived mesenchymal stem cells undergo molecular changes after a single passage in culture. Sci Rep. 2024 May 29;14(1):12396. Epub 2024/05/30. doi:10.1038/s41598-024-63009-8. Cited in: Pubmed; PMID 38811646.

9. Livak KJ, Schmittgen TD. Analysis of relative gene expression data using real-time quantitative PCR and the 2(-Delta Delta C(T)) Method. Methods. 2001 Dec;25(4):402-8. Epub 2002/02/16. doi:10.1006/meth.2001.1262. Cited in: Pubmed; PMID 11846609.
