# Supplementary material for: Pharmacological inhibition of sclerostin protects bone from B‐cell acute lymphoblastic leukemia‐mediated destruction
Source: Hemasphere. 2026 Mar 31;10(4):e70355. doi: 10.1002/hem3.70355 (PMC13103727; doi:10.1002/hem3.70355)
Supplement: Supplementary file 2 — Hemasphere Supplemental Figures and Legends. [file HEM3-10-e70355-s002.docx]

**Supplemental Figures**

Pharmacological inhibition of sclerostin protects bone from B-cell acute lymphoblastic leukemia-mediated destruction

**
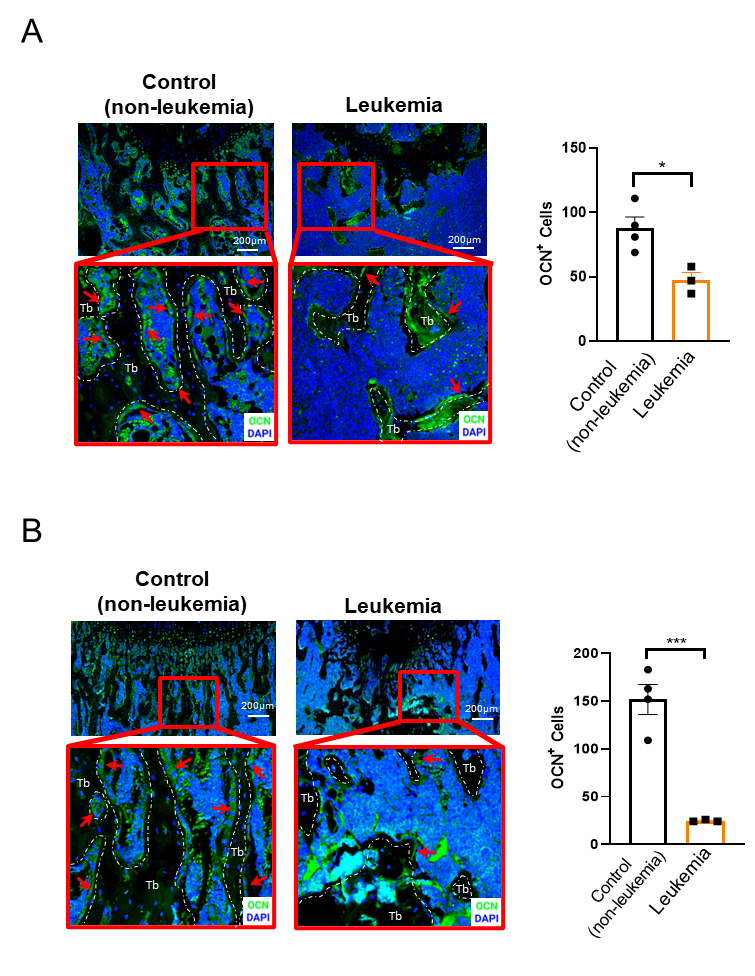
**

**Supplemental Figure 1. B-ALL progression contributes to a reduction in the number of osteocalcin (OCN^+^) bone-lining cells.** (A) Representative images followed by quantitative analysis showing the average number of OCN^+^ bone-lining cells (per field of 0.5mm below the growth plate) in control (non-leukemic) mice and patient-derived xenografts bearing ALL-84 B-ALL (OCN staining = green and DAPI nuclear staining = blue). (B) Representative images followed by quantitative analysis showing the average number of OCN^+^ bone-lining cells in control (non-leukemic) mice and syngeneic mice bearing PER-M60 BCR-ABL1^+^ B-ALL. Datapoints represent biological samples (n=3-4 per group). Error bars represent mean ± SEM. *p<0.05, ***p<0.001.

**
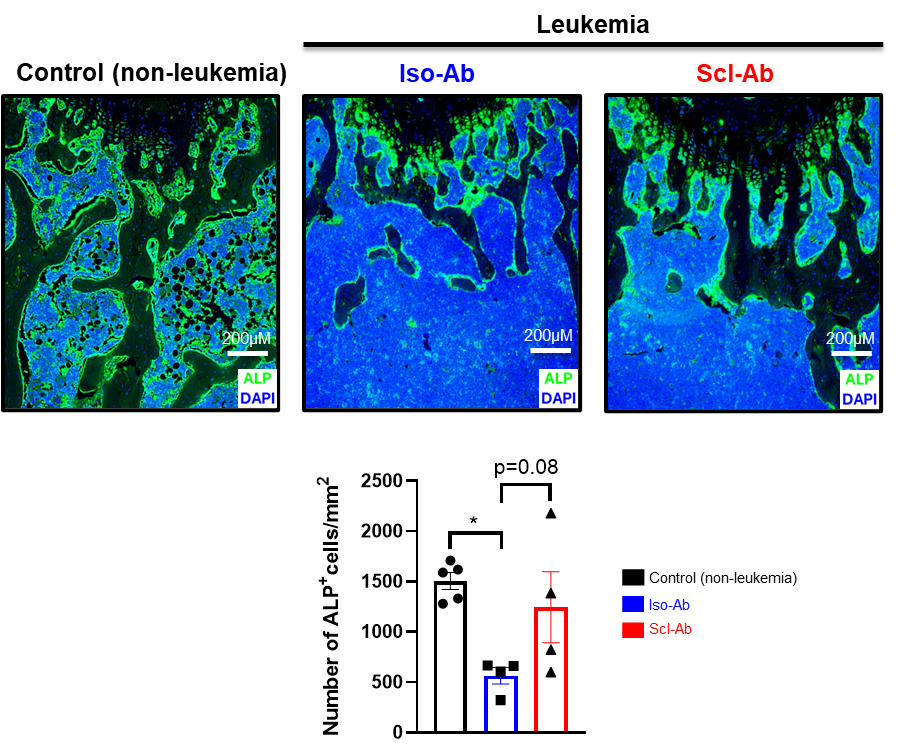
**

**Supplemental Figure 2. Immunofluorescence staining showing the expression of alkaline phosphatase (ALP) in the trabecular bones of leukemia-bearing mice treated with Scl-Ab.** Mice were treated with Scl-Ab or Iso-Ab for 2 weeks, starting at a bone marrow disease burden of 11.64±2.53%. Representative images followed by quantitative analysis showing the average number of ALP^+^ cells per mm^2^ (per field of 0.5mm below the growth plate) in non-leukemic control mice and ALL-84 leukemic mice treated with Scl-Ab or Iso-Ab (ALP staining = green and DAPI nuclear staining = blue). Datapoints represent biological samples (n=4-5 per group). Error bars represent mean ± SEM. *p<0.05.

**
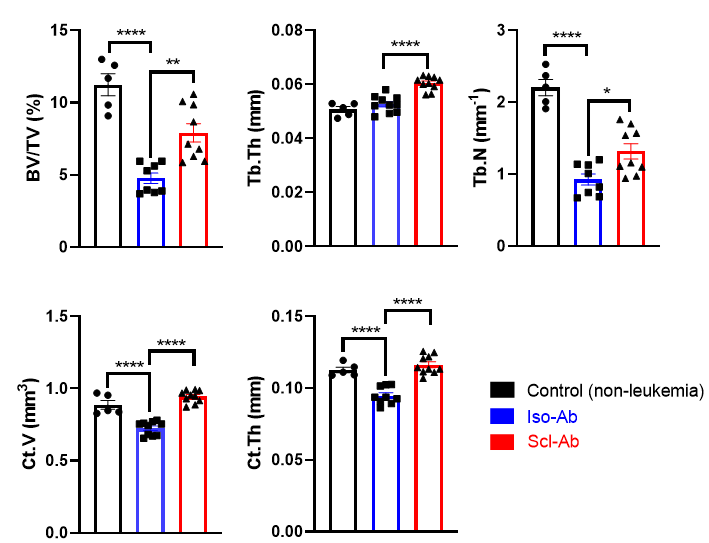
**

**Supplemental Figure 3. Scl-Ab treatment improves bone parameters in syngeneic mice bearing PER-M60 BCR-ABL1^+^ B-ALL.** Mice received continuous treatments with Scl-Ab or Iso-Ab control antibody commencing 3 days following PER-M60 leukemia cell injection until they succumbed to disease. Quantifications of trabecular bone volume per tissue volume (BV/TV), trabecular thickness (Tb.Th), trabecular number (Tb.N), cortical volume (Ct.V) and cortical thickness (Ct.Th). Datapoints represent biological samples (n=5-10 per group). Error bars represent mean ± SEM. *p<0.05, **p<0.01, ****p<0.0001.

**
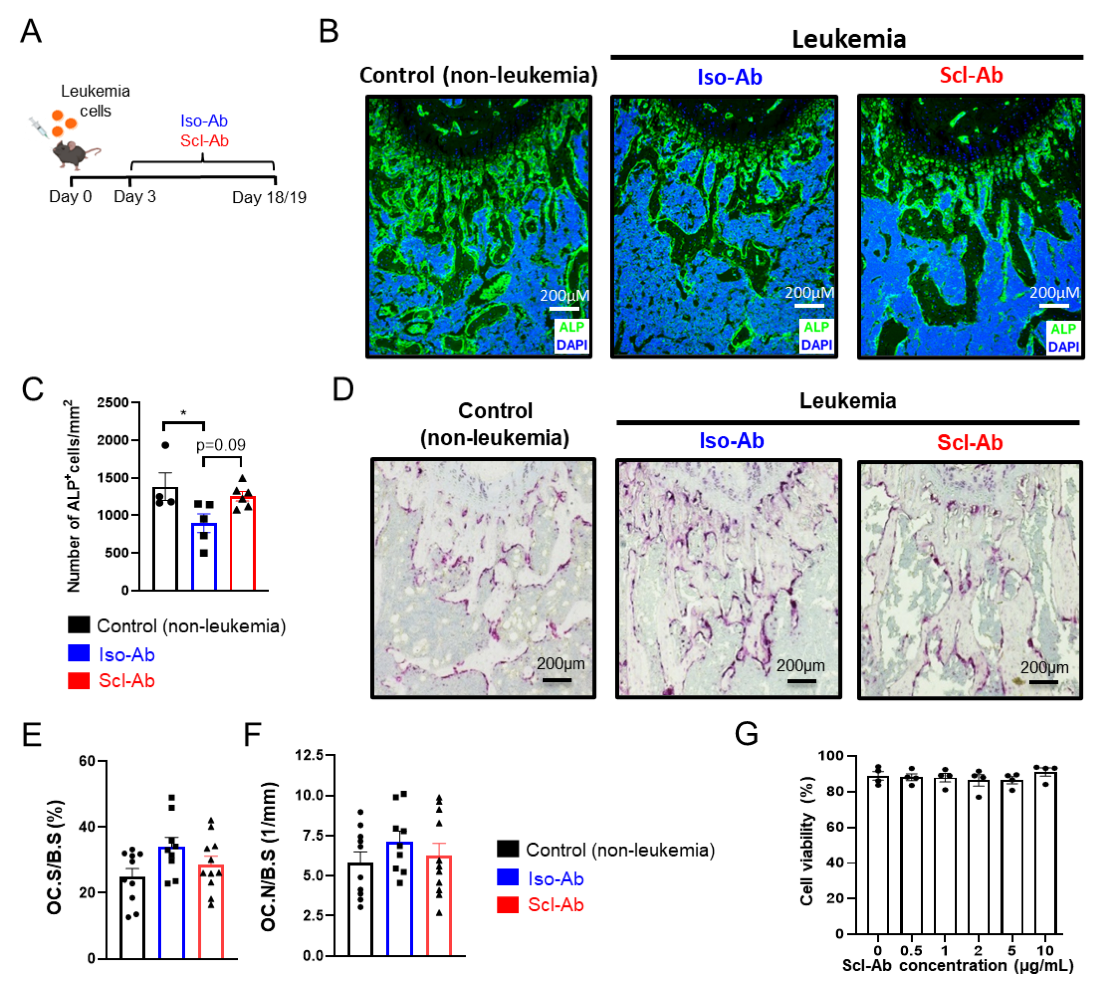
**

**Supplemental Figure 4. Effect of Scl-Ab treatment on alkaline phosphatase (ALP^+^) cells, osteoclast number and viability of BCR-ABL1^+^ B-ALL cells.** (A) Schematic of treatment. Mice were treated with Scl-Ab or Iso-Ab for 2 weeks, starting on day 3 post-PER-M60 leukemia cell injection. Mice were sacrificed on day 18/19 and bones were extracted for histology. (B) Representative Immunofluorescent images followed by (C) quantitative analysis showing the average number of ALP^+^ cells per mm^2^ (per field of 0.5mm below the growth plate) in healthy control (non-leukemic) mice and PER-M60 leukemic mice treated with Scl-Ab or Iso-Ab (ALP staining = green and DAPI nuclear staining = blue). Datapoints represent individual biological samples (n=4-6). (D) Representative images showing tartrate-resistant acid phosphatase-stained metaphyseal region of distal femurs extracted from healthy control mice, leukemic mice treated with Iso-Ab and leukemic mice treated with Scl-Ab. Quantitative analyses of (E) osteoclast surface per bone surface (OC.S/B.S) and (F) number of osteoclasts per bone surface (OC.N/B.S) in the control and treatment groups. Datapoints represent random fields of view (total of 9-11 per group) taken from 4 biological samples, per group. (G) Viability of PER-M60 cells following a 3-day *in vitro* culture with Scl-Ab at 0.5, 1, 2, 5 and 10 µg/mL. Datapoints represent technical replicates (n=4). Error bars represent mean ± SEM. *p<0.05.

**
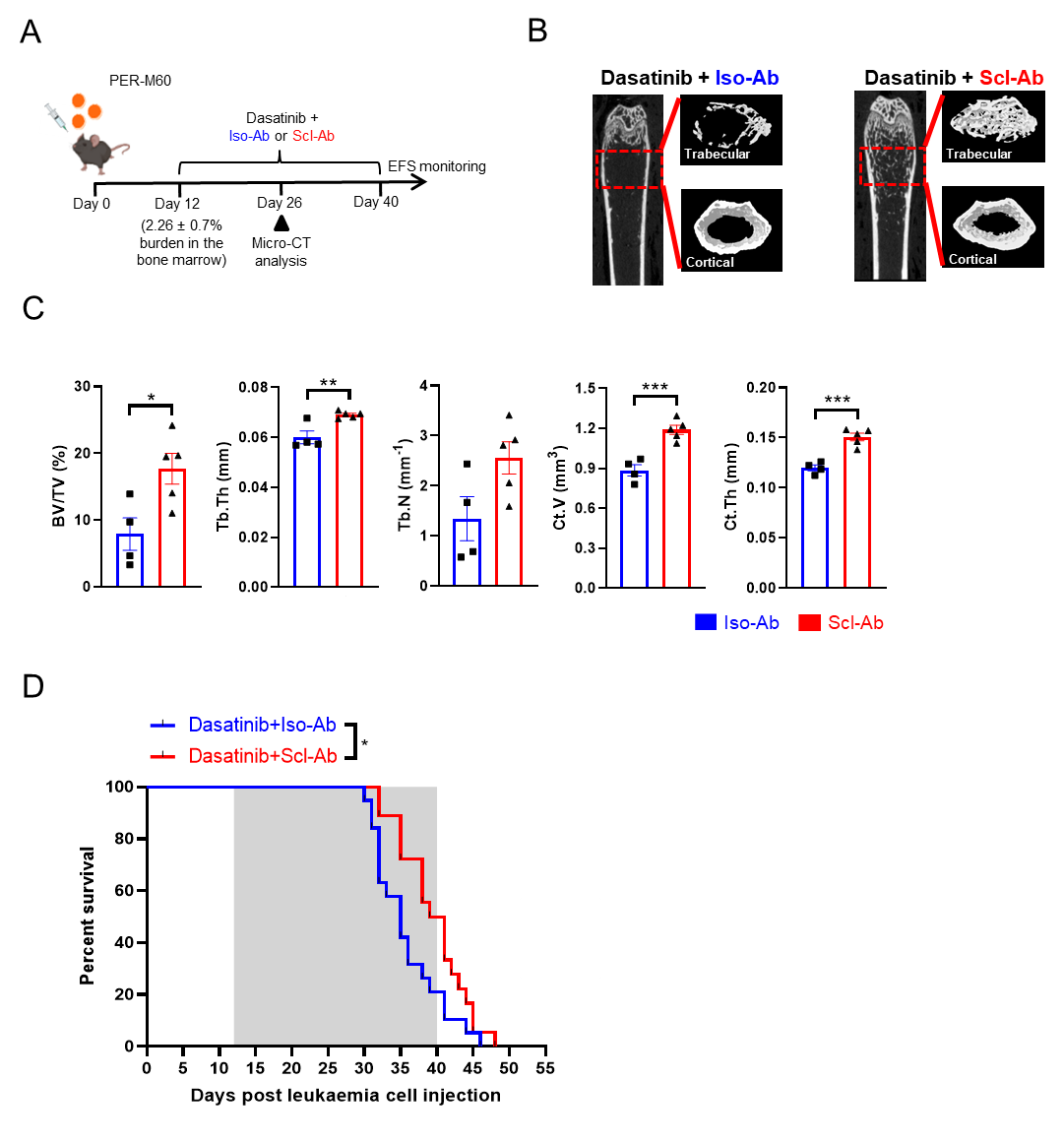
**

**Supplemental Figure 5. Combination of dasatinib and Scl-Ab treatment improves bone parameters and extends survival in syngeneic mice bearing PER-M60 leukemia.** (A) Schematic of treatment. Mice were treated with dasatinib for a duration of 4 weeks, starting on day 12-post leukemia cell injection when the bone marrow disease burden was 2.26 ± 0.7%. Scl-Ab or Iso-Ab were given during the first 2 weeks of dasatinib treatment. Micro-CT analysis was conducted on day 26 (end of Scl-Ab/Iso-Ab treatment) and event-free survival monitoring was performed throughout the experiment. (B) Representative 3-dimensional reconstructed micro-CT images showing the distal femur compartments of leukemic mice treated with dasatinib + Iso-Ab or dasatinib + Scl-Ab. (C) Quantification of trabecular bone volume per tissue volume (BV/TV), trabecular thickness (Tb.Th), trabecular number (Tb.N), cortical volume (Ct.V) and cortical thickness (Ct.Th). Datapoints represent biological samples (n=4-5 per group). (D) Kaplan-Meier survival curve showing the survival of PER-M60 leukemia-bearing mice treated with dasatinib in combination with Scl-Ab (n=18) or Iso-Ab (n=19). The grey shaded areas indicate the treatment periods. Error bars represent mean ± SEM. *p<0.05, **p<0.01, ***p<0.001.

**
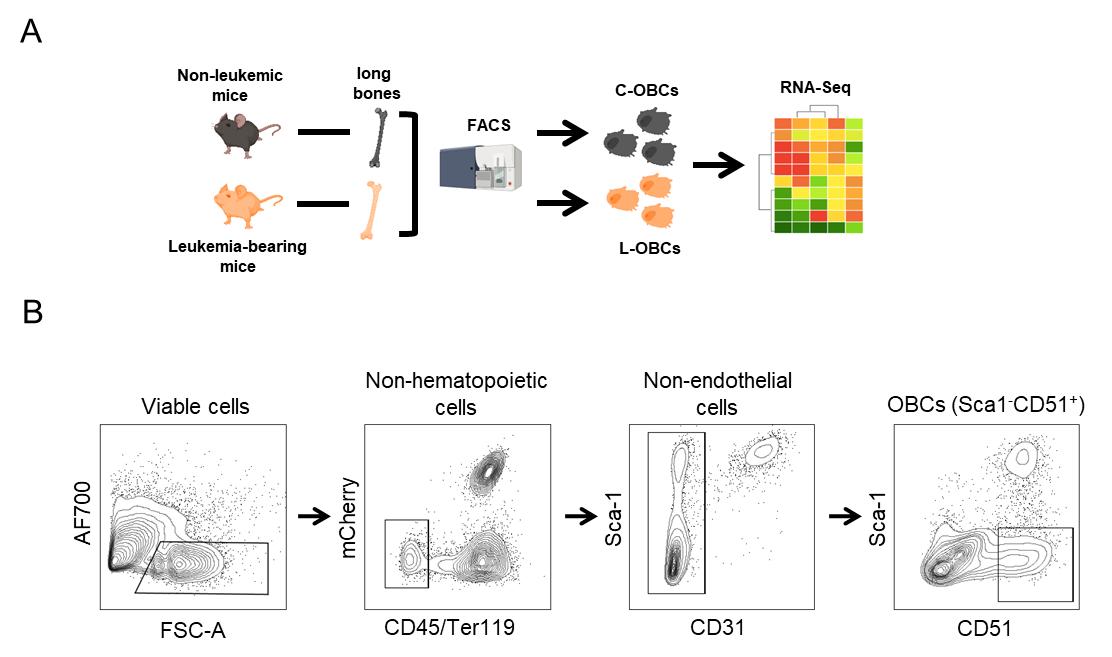
**

**Supplemental Figure 6. Isolation and processing of leukemia-associated osteoblastic cells (L-OBCs) and control osteoblastic cells (C-OBCs) for RNA sequencing.** (A) Schematic diagram showing the process of extracting OBCs from the long bones of healthy (non-leukemic mice) and syngeneic mice bearing PER-M60 BCR-ABL1^+^ B-ALL for RNA sequencing. C-OBCs and L-OBCs were sorted via fluorescence-activated cell sorting (FACS). (B) Gating strategy of flow cytometry/FACS. Viable OBCs are defined as non-leukemic (mCherry^-^) cells expressing AF700^-^, CD45/Ter119^-^, CD31^-^, Sca1^-^, CD51^+^.

**
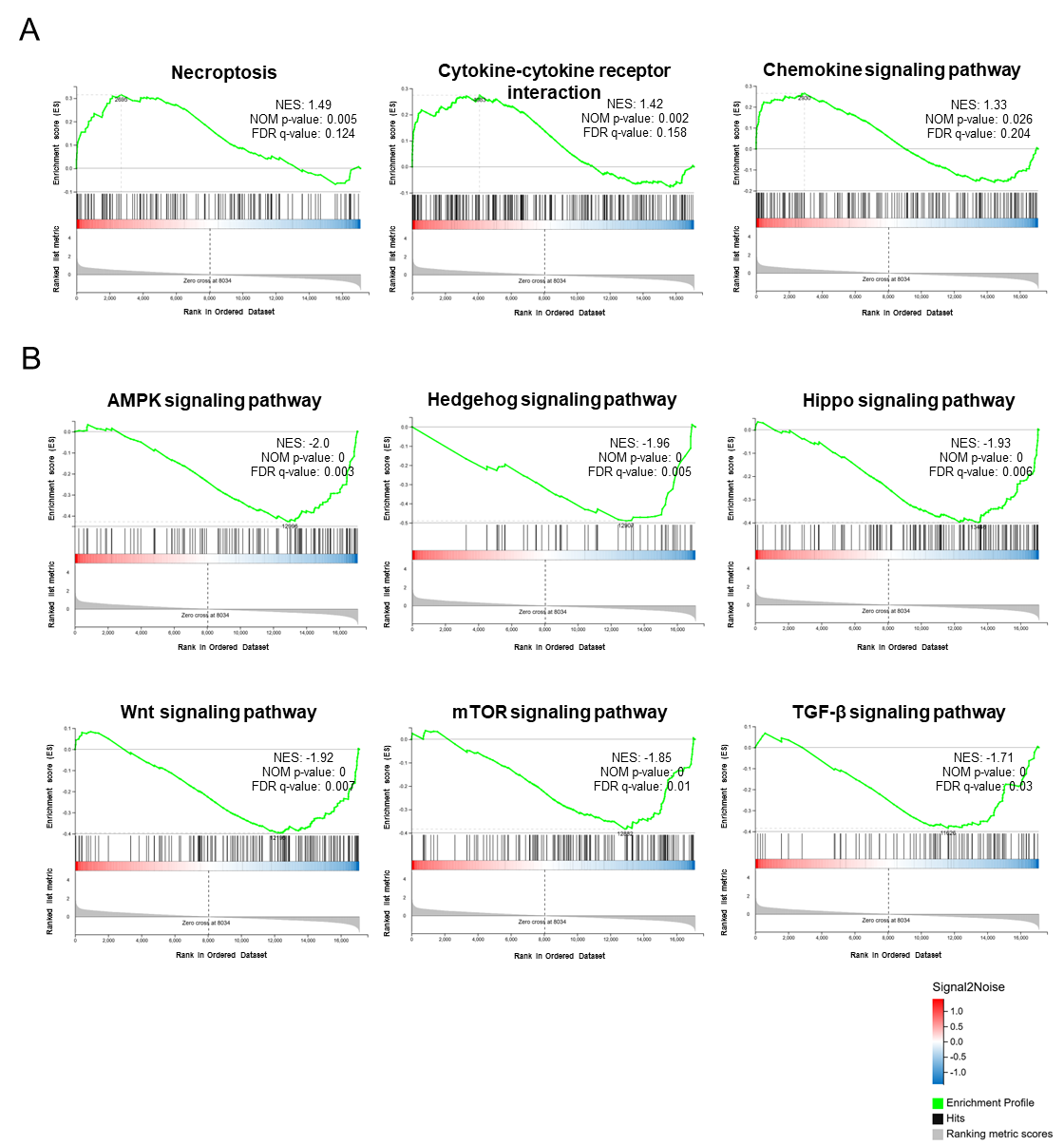
**

**Supplemental Figure 7. Gene set enrichment analysis (GSEA) of primary control osteoblastic cells and leukemia-associated osteoblastic cells (L-OBCs) using the Kyoto Encyclopedia of Genes and Genomes (KEGG) database.** (A) Gene sets upregulated in L-OBCs (necroptosis, cytokine-cytokine receptor interaction and chemokine signaling pathway) and (B) Gene sets downregulated in L-OBCs (AMPK, Hedgehog, Hippo, Wnt, mTOR and TGF-β signaling pathways). A |normalized enrichment score (NES)|≥ 1 or ≤ -1, nominal (NOM) p-value ≤ 0.05 and false discovery rate (FDR) q-value ≤ 0.25 were used as threshold values.

**
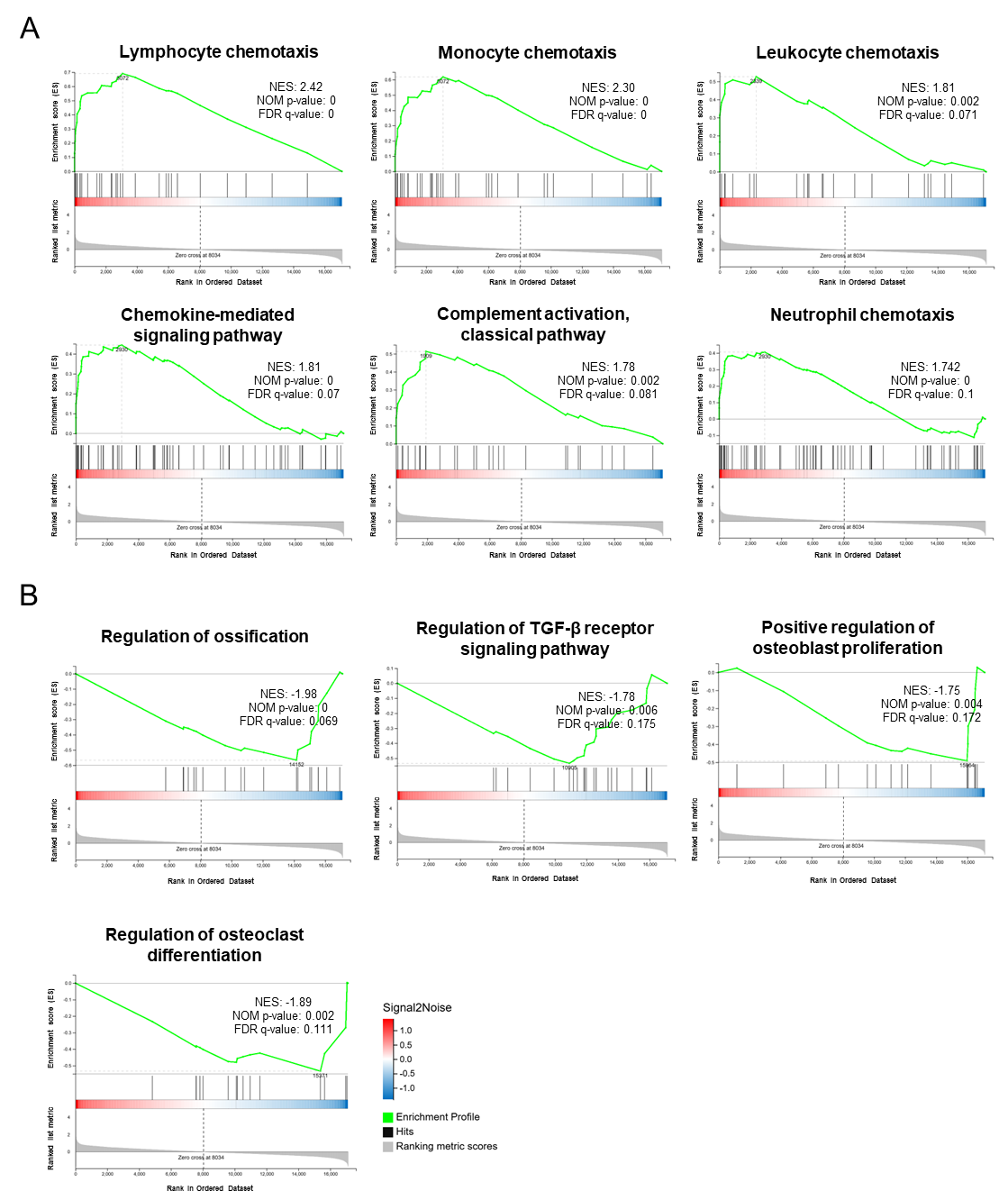
**

**Supplemental Figure 8. Gene set enrichment analysis (GSEA) of primary control osteoblastic cells and leukemia-associated osteoblastic cells (L-OBCs) using the Gene Ontology Biological Processes (GO-BP) domain.** (A) Gene sets upregulated in L-OBCs (lymphocyte chemotaxis, monocyte chemotaxis, leukocyte chemotaxis, chemokine-mediated signaling pathway, complement activation, classical pathway, and neutrophil chemotaxis) and (B) Gene sets downregulated in L-OBCs (regulation of ossification, regulation of TGF-β receptor signaling pathway, positive regulation of osteoblast proliferation and regulation of osteoclast differentiation). A|normalized enrichment score (NES)|≥ 1 or ≤ -1, nominal (NOM) p-value ≤ 0.05 and false discovery rate (FDR) q-value ≤ 0.25 were used as threshold values.

**
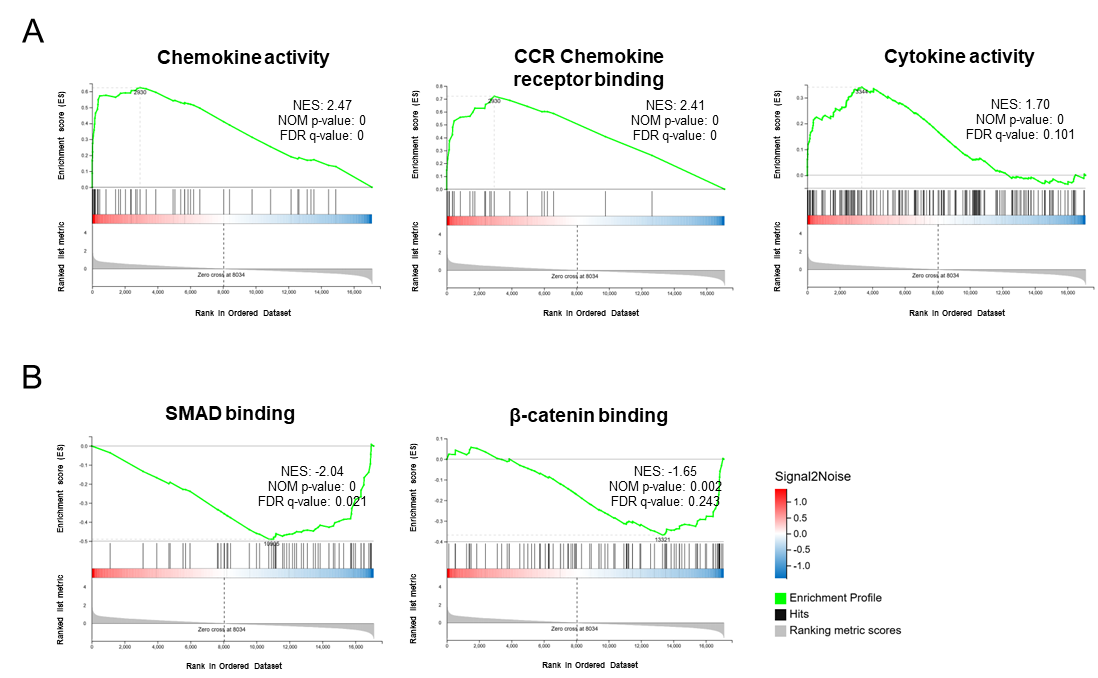
**

**Supplemental Figure 9. Gene set enrichment analysis (GSEA) of primary control osteoblastic cells and leukemia-associated osteoblastic cells (L-OBCs) using the Gene Ontology Molecular Function (GO-MF) domain.** (A) Gene sets upregulated in L-OBCs (chemokine activity, CCR chemokine receptor binding and cytokine activity) and (B) Gene sets downregulated in L-OBCs (SMAD binding and β-catenin binding). A|normalized enrichment score (NES)|≥ 1 or ≤ -1, nominal (NOM) p-value ≤ 0.05 and false discovery rate (FDR) q-value ≤ 0.25 were used as threshold values.

**
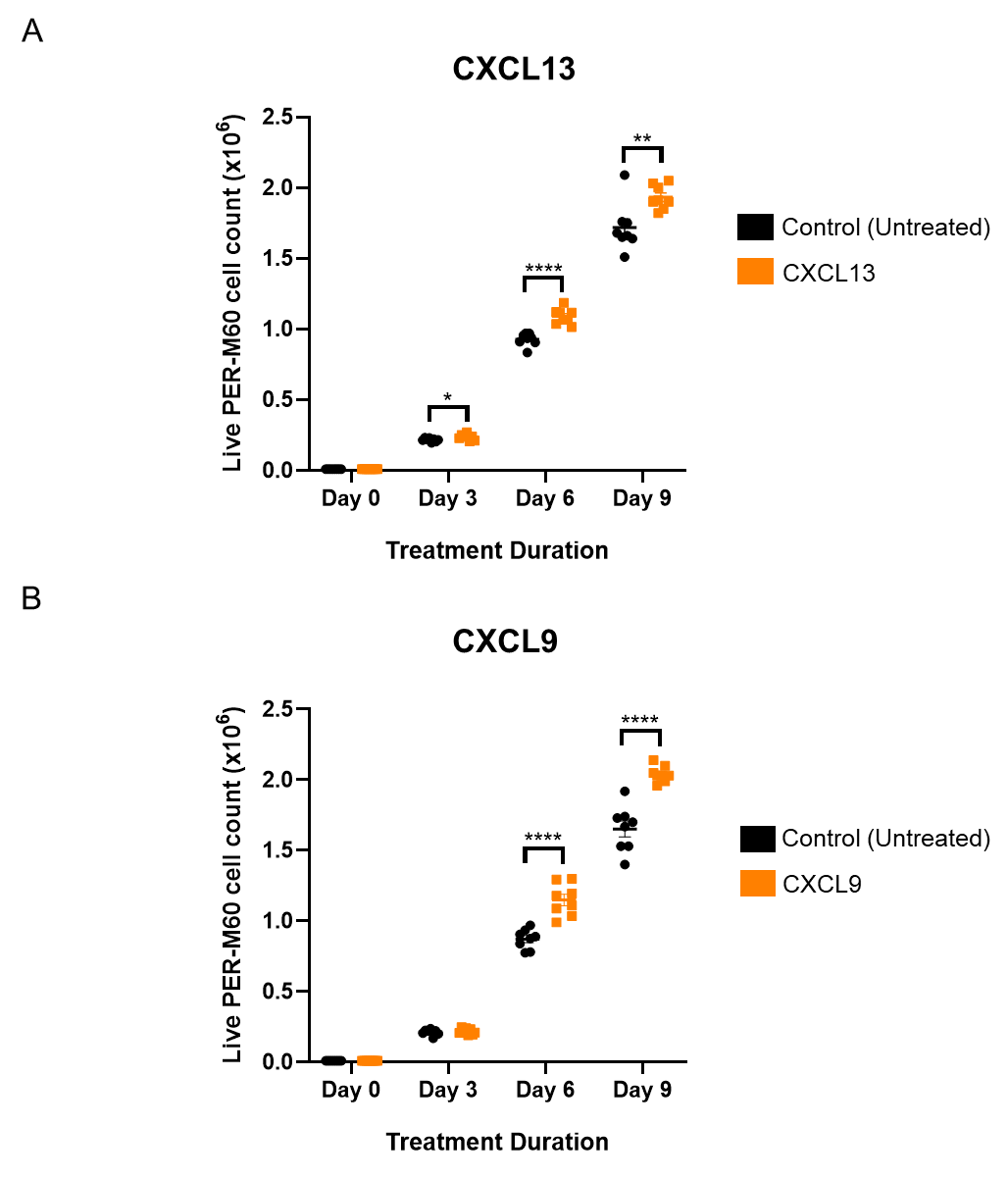
**

**Supplemental Figure 10.** **Recombinant mouse CXCL13 and CXCL9 proteins promote an increase in the number of PER-M60 leukemic cells *in vitro*.** PER-M60 cells were cultured in the presence of 200ng/mL recombinant mouse (A) CXCL13 or (B) CXCL9 for a duration of 3, 6 and 9 days. Untreated PER-M60 cells were used as control. Datapoints represent live PER-M60 cell count from technical replicates (n=8 per group). Error bars represent mean ± SEM. *p<0.05, **p<0.01, ****p<0.0001.
